# Barriers encountered during the implementation of a policy guideline on the vaccination of health care workers during the 2013–2014 measles outbreak in the Netherlands: a qualitative study

**DOI:** 10.1186/s13104-015-1756-x

**Published:** 2015-12-14

**Authors:** Stephanie Jessica Borggreve, Aura Timen

**Affiliations:** Rijksinstituut voor Volksgezondheid en Milieu, Antonie van Leeuwenhoeklaan 9, 3721 MA Bilthoven, The Netherlands

**Keywords:** Measles, Vaccination, Health care workers, Public Health Policy

## Abstract

**Background:**

In 2013 the Netherlands faced a measles epidemic, during which more than 2600 individuals were infected, including 19 health care workers (HCW). Vaccinating health care workers can lead to benefits on both the individual and public health level, underscoring the need for HCW vaccination. In June of 2013 the Dutch National Institute for Public Health and the Environment (RIVM) developed a measles guideline (MG) that advised Dutch hospitals to strengthen their policies concerning measles vaccination of HCWs. A key problem with guidelines, however, is adherence, which can be due to several barriers. The objective of this research was to identify the barriers that Dutch hospital professionals encountered during the implementation of this policy guideline, in order to improve the implementation of similar policies in the future.

**Methods:**

In-depth interviews (n = 9) were conducted with 12 hospital health care professionals involved with prevention and control of communicable diseases. These participants represented ten different Dutch hospitals located in eight of the twelve different provinces. Participants were asked about their experiences during the 2013–2014 measles epidemic regarding infection prevention measures, including vaccination of HCWs, with a specific focus on barriers to the implementation of the RIVM guideline.

**Results:**

The implementation of the MG was impeded by several (types of) barriers. First, barriers were found related to knowledge and attitude, and included lack of agreement, barriers associated with leadership and issues related to evidence-based decision making. Second, barriers related to characteristics of the guideline, mostly related to unclear or missing guideline content. Finally, contextual and social factors such as human and financial resources, belief systems, physical facilities and technical support, and national views on vaccination policies also play an important role in policy implementation.

**Conclusions:**

This study has provided valuable insights into the barriers infection prevention specialists encounter during the implementation of new policies concerning vaccination of HCWs in times of a major outbreak. Moreover, this study exposed the complexity and breadth of barriers that are of importance when implementing vaccination policies in the hospital setting. In order to improve the implementation of similar policies in the future, guideline developers and health care providers and administrators alike should aim to eliminate or minimise these identified barriers by taking into account the suggestions made by the authors.

## Background

Measles caused approximately 158.000 deaths globally in 2011, mostly among young children [1]. Measles is caused by a virus that belongs to the family of Paramyxoviridae viruses and is typically characterized by fever, cough, conjunctivitis and a rash that spreads from the face to the rest of the body [2]. Complications of measles include otitis media, pneumonia and encephalitis [3].

The MMR (measles, mumps, rubella) vaccination coverage in the Netherlands is high (>95 %) and in the general population herd-immunity protects those who are not vaccinated. Despite this high vaccination coverage in the general population, there are areas where religious orthodox protestant individuals refuse vaccination. In these regions (the so-called “bible belt”) the mean vaccination coverage is approximately 60 % [4]. This group comprises approximately 250,000 persons, mostly living in an area that stretches from the southwest to the northeast of the country with shared educational and social activities. These areas have played a substantial role in past measles epidemics and recently, again, during the outbreak of 2013–2014. The incidence of measles decreased dramatically in the Netherlands after the introduction of measles vaccination in 1976, with an average of 10–15 notifications yearly. However, major outbreaks occurred in the orthodox population every 10–12 years [5]. During the 2013–2014 epidemic more than 2600 patients were diagnosed with measles, of which one case was fatal [6]. Furthermore, spread of the infection from the Netherlands contributed to a local epidemic in Alberta, Canada [6].

Due to the severe nature of the disease, 182 individuals were hospitalised with measles infection during the Dutch epidemic [6]. Since measles is not a common disease in non-endemic countries, it may not be directly recognized in the patient, which may result in a failure to implement appropriate isolation precautions and an increased risk of nosocomial transmission [7–9]. The latter holds true since measles is one of the most highly contagious communicable diseases, in which droplet transmission occurs before the onset of rash, thereby exposing susceptible individuals to the infection [8, 10]. In the healthcare setting, measles can lead to severe morbidity and mortality because hospitalised patients are highly vulnerable to infection [11].

In addition to patients, health care workers (HCWs) are at risk of becoming infected. HCWs in hospitals have a 2–19 times increased risk of infection compared to adults in the general population [12–14]. Furthermore, health care personnel under the age of 30 are more susceptible to measles infection compared to older health care personnel [15, 16]. Therefore, health care workers are of significant importance in disease transmission, since they can be infected themselves and pose a risk to their patients. Together, the individual and public health burden of measles in the healthcare setting underscores the importance of protecting health care personnel from infection through vaccination. Both the personal benefit to the HCW that results from a decreased risk of infection benefits all and helps ensure less disruption in health care delivery.

During the Dutch epidemic, 19 healthcare workers were infected with measles, of whom 12 were unvaccinated and 4 had been vaccinated with only one dose of the vaccine [6]. In June of 2013, at the beginning of the outbreak the Dutch National Institute for Public Health and the Environment (Rijksinstituut voor Volksgezondheid en Mileu, RIVM) issued guidance on prevention and control of measles by targeting HCWs in hospitals. This “Advice on protection against measles in healthcare settings” (the Measles Guideline, MG) focused on screening- and immunisation policies, post-exposure policies, and control measures regarding patients with measles [17].

A key problem that arises with the implementation of guidelines is adherence. Cabana et al. [18] conducted a literature study into barriers to clinical practice guideline adherence among physicians. A barrier was defined as “any factor that limits or restricts complete physician adherence to a guideline” [18]. The authors identified a framework comprised of seven main barriers to adherence: lack of awareness; lack of familiarity; lack of agreement; lack of self-efficacy; lack of outcome expectancy; inertia of previous practice; and environmental factors. Cahill et al. [19] conducted semi-structured interviews based on the Cabana framework with Intensive Care Unit (ICU) medical directors, nurse managers, physicians, clinical nurse educators, dieticians and bedside nurses to identify factors that form barriers to adherence to guidelines. While confirming the barriers identified by Cabana et al. [18], Cahill et al. also found that cultural aspects of the organisation such as leadership style, communication and teamwork are additional factors that influence guideline adherence [19].

Adherence to guidelines has been subject to various studies in the Dutch setting. The most relevant of these studies in this context was conducted by Timen et al. [20], who studied barriers among Dutch key professionals in communicable disease outbreak control during crisis situations using the framework of Cabana et al. [18]. Through questionnaires and in-depth interviews Timen et al. found four barriers to adherence to guidelines to be of major importance: (1) “No concrete targets for performance to measure the effectiveness of the measures”; (2) “Control measures are worded with insufficient urgency or definition”; (3) “Crucial instructions within control measures (concerning isolation, diagnostics, and treatment) are not clear or easily identifiable for each profession” and (4) “Measures regarding the use of personal protective equipment are inadequate or not timely” [20].

The objective of this research is to identify the barriers that hospital professionals encountered when implementing of the MG during the outbreak, in order to improve the implementation of similar policies in the future.

## Methods

This research was conducted in the Netherlands between February and July of 2014. Formal ethical consent was deemed unnecessary according to national regulations described in the “General procedure regarding trials that are subject to the WMO”, RIVM,

CIb-PRO-1003 (ID 013683) 2014, and the Dutch Law entitled “Wet Medisch-wetenschappelijk Onderzoek met mensen (WMO), July 2012. We applied the consolidated criteria for reporting qualitative research (COREQ, see Additional file) and adhered to RATS guidelines for reporting qualitative research [21].

### The measles guideline (MG)

According to the guideline, the hospital should review the immune status of employees of high-risk departments (paediatrics, neonatology, obstetrics and the maternity ward, internal medicine, intensive care, and emergency departments). This applies not only to those with direct patient contact, but also to (medical) auxiliary personnel, such as radiology- and physiotherapy departments, as well as cleaning and administrative staff, nutritionists, and (para-) medical students working in these departments. Employees not protected against measles should be offered vaccination. Specific attention should be paid to pregnant women and immunocompromised individuals since vaccination in these two populations is contra-indicated.

The guideline provides clear definitions of individuals sufficiently protected (born before 1965 or between 1965 and 1985 and positive measles history), moderately protected (born after 1975 with only one received measles vaccination) and not protected (those who do not meet the criteria for moderately or sufficiently protected). HCWs moderately protected should receive one dose of MMR-vaccine, whereas those unprotected should receive two doses, at least 1 month apart. Contra-indications for vaccination are pregnancy and immune suppression.

Infection prevention measures for patients are specified in the guideline. Patients with suspected measles infection must be placed in strict isolation during 7 days following onset of the rash [22]. Healthcare workers must wear filtering face piece (FFP) 2 masks while caring for those patients. Initially, the policy regarding isolation and face mask usage differed from the ruling guidelines of the Dutch Working Party on Infection Prevention (WIP), which advised for droplet precautions and FFP 1 mask. Later, the discrepancies were solved [22].

Finally, the MG describes post-exposure measures for both HCWs and patients. Those sufficiently protected do not need to undergo further measures. Those moderately and not protected must receive MMR-vaccination as soon as possible after contact, together with laboratory testing for antibodies. A second dose of the MMR-vaccine should be administered 1 month later. HCWs with an increased risk of complications (i.e. pregnant women and immunosuppressed individuals) should have laboratory testing of antibody levels and can be offered immunoglobulins, after consultation with the treating physician. When no protective antibodies are found, exposed HCWs must be prohibited from working for a period of 5–18 days post-exposure [17].

### Sampling of participants

In-depth semi-structured interviews were conducted to understand barriers encountered by hospital health care professionals responsible for implementing the MG. As hospitals may allocate the implementation tasks to various categories of professionals, infection prevention specialists, occupational health physicians, microbiologists and managers of infection prevention departments were selected to comprise the research sample.

In the Netherlands there are 84 general and 8 academic hospitals [23]. For the sampling process the country was divided into three areas: north, middle and south. This distinction is used by the Dutch government on the presumption that these three regions each contain approximately an equal number of inhabitants [24]. For each region, two hospitals with the highest number of beds and two with the lowest number of beds were selected for participation [25]. For each region at least one large and one small hospital were selected where measles occurred during the 2013–2014 epidemic. Incidence of measles was identified from the national measles surveillance data base [26]. A 13th hospital was included in the sample due to its large number of measles cases (according to the preliminary database). These 13 hospitals were located in eight of the twelve Dutch provinces.

### Data collection

Interview invitations were sent by post to the boards of 13 hospitals and were followed up by a telephone call. The board was requested to forward the interview invitation to the hospital professional deemed most suitable for participation. The letter provided background information on the research and procedure, as well as a description of the requirements hospital professionals should meet in order to participate in this research. The most important requirement for participation was having been directly involved in developing/implementing policies concerning measles during the 2013–2014 epidemic.

Interviews were conducted in Dutch, on site or by telephone by one researcher (S.B.), lasted 20–40 min and were audiotaped. Before the interview, informed consent was obtained verbally from the interviewees. An interview guide was used that was developed based on the validated framework of Cabana [18] to standardise reporting of barriers, to which elements from several other barrier studies were added [19, 20]. Our framework thus included the seven barriers derived from Cabana et al. [18] plus three from Cahill et al. [19], and four from Timen et al. [20]. New elements brought up by participants were coded post hoc.

### Data analysis

The interviews were transcribed verbatim by one researcher (S.B.), making use of the F4 program, version 4.2 (F4, dr.dresingamp, pehl GmbH, Germany). Transcripts were coded with the Atlas T.I. program, version 7.1.3 (ATLAS.ti GmbH, Berlin) according to a previously developed coding scheme, which was based on the literature study and the interview guide. Elements that were not included in the original coding scheme, but were mentioned by participants, were awarded their own new code during the data analysis stage. Similarly coded text elements were grouped together in families and were summarised and illustrated by interviewee quotes. Two researchers separately coded the data in order to prevent bias. Discrepancies were solved by discussion between the two researchers. The main goal was to capture the whole spectrum of possible barriers rather than quantifying their relative importance.

## Results

Three hospitals did not respond to the invitation to participate in this research, despite extensive follow-ups. In total, nine interviews were conducted with hospital professionals, representing a total of ten hospitals (one participant represented two hospitals). Of these ten hospitals, six had measles cases in their hospital at the moment of sampling; four had none at that time. Because of the relatively small sample size, differences between the encountered barriers were not further explored statistically. With these nine interviews saturation of data was reached, i.e. no new information would be obtained during additional interviews. Seven interviews were conducted on site; two were conducted by telephone. In three instances two professionals from a given hospital were interviewed together for a total of 12 participating professionals. Interviewees represented two academic centres and eight non-academic hospitals. Academic centres have an additional task in performing biomedical research. The professional background of the participants was: infection prevention specialist (n = 5), occupational health physician (n = 3), microbiologist (n = 2) and manager of the infection prevention department (n = 2).

A total of 22 different barriers to the implementation of the guideline were discussed, of which 17 were encountered by at least one of the professionals during this outbreak. Discussion of these barriers was partly prompted by the interviewer and partly arose spontaneously from the participants. The barriers were categorised into three main categories, based on the aforementioned frameworks. The first category refers to barriers related to knowledge and attitude, and mainly focuses on the individuals implementing the guideline. The second category is composed of barriers related to characteristics of the guideline itself. The final category focuses on barriers related to contextual and social factors that surround the implementation of the guideline. Barriers encountered by at least four participants are discussed in detail below. Remaining barriers experienced by less than 4 participants are listed in a separate category for the purpose of result completeness. Table 1 provides an overview of all barriers and the number of participants encountering these barriers.

**Table 1:** Overview of the number of explored and encountered barriers

Category	Barrier	Number of participants
Barriers related to knowledge and attitude	Lack of agreement [18]	10
	Leadership [19]	7
	Issues related to evidence-based decision making [new]	6
	Communication [19]	4
	Unclear division of labour [19], based on “teamwork” [19]	3
	Lack of outcome expectancy [18]	2
	Inertia of previous practice [18]	1
	Lack of awareness [18]	0
	Lack of familiarity [18]	0
	Lack of self efficacy [18]	0
Total endorsements in this domain		33
Total number of explored barriers in this domain		10
Total number of encountered barriers in this domain		7
Barriers related to characteristics of the guideline	Unclear guideline content [new]	10
	“Crucial instructions within control measures (concerning isolation, diagnostics, and treatment) are not clear or easily identifiable for each profession” [20]	4
	Unclear phrasing of the guideline [new]	3
	“Control measures are worded with insufficient urgency or definition” [20]	2
	“Lack of concrete targets for performance to measure the effectiveness of the measures” [20]	0
	“Measures regarding the use of personal protective equipment are inadequate or not timely” [20]	0
Total endorsements in this domain		19
Total number of explored barriers in this domain		6
Total number of encountered barriers in this domain		4
Barriers related to contextual and social factors	Barriers related to timing [17, 18]	7
	Barriers related to finances and (high) working pressure [18]	6
	Barriers related to physical aspects [18]	6
	Lack of clear national views on vaccination policies [new]	5
	Presence of multiple guidelines [new]	3
	Barriers related to (hospital) culture [19]	2
Total endorsements in this domain		29
Total number of explored barriers in this domain		6
Total number of encountered barriers in this domain		6
Total number of barriers explored		22
Total number of barriers encountered by participants		17

Barriers explored and encountered by the participants (n = 12). The reference is provided between brackets

### Barriers related to knowledge and attitude

#### Barrier: lack of agreement (n = 10)

Lack of agreement was mainly found to be of importance on the level of the individual HCWs, who did not always agree with the new policies. Nearly all participants indicated to have experienced resistance from HCWs to vaccination policies (n = 10). This was either direct resistance to the policy or resistance displayed by not responding to communication. Main reasons for direct resistance included religious reasons, fear of side-effects, wanting to get pregnant and therefore not being able get to vaccinated, not wanting to share immune status or “just not wanting it” [participant. 4]. As one participant indicated:

“This is a vaccination you get for somebody else, and not for yourself, and that is a completely different motivation.” [participant 12]

Most participants faced difficulties when HCWs were requested to disclose their immune status. Either some HCWs did not respond at all, or HCWs not immune to measles sometimes refused vaccination.

#### Barrier: issues related to evidence-based decision making (n = 6)

The Cabana model originally included a category that relates to a lack of self-efficacy, i.e. the participants doubting whether they were able to implement this new policy. In the current study participants’ doubts were related to the evidence underlying their decision-making processes. First, the participants wondered whether their efforts justify the risks associated with measles and indicated that the fear existed to make a lot of effort “for nothing”, as worded below:

“Should we start running again, and for what? A lot of work.” [participant 3]

Not having access to the actual number of cases during an outbreak specific to different regions/hospitals was a barrier to taking action as it is difficult to assess if you should worry and take action or wait a little longer.

#### Barrier: leadership (n = 7)

Lack of strong leadership was considered to impair the success of the vaccination policies. Five participants indicated that the role of the hospital board was rather limited and was restricted to being informed only. Only two participants acknowledged that the board played a significant role and was accountable for hospital policy. Strong leadership is a key facilitator when the vaccination policy is issued on behalf of the hospital board. Participants indicated that full cooperation of the hospital board was highly instrumental in assuring cooperation from other departments. Furthermore, when communication is top-down HCWs get a clear signal and better understand the measures necessary to handle a given situation. At the same time the responsibility does not lie solely with single individuals specialised in infection prevention.

“I do believe that it [policy] should be carried by the layer above and they provide feedback to the board. The HCW always finds it important to know where it exactly comes from. So if you look at it like that it is important that it is carried, also by the board and the underlying management staff.” [participant 9]

In one hospital, however, the involvement of the board was experienced as too overbearing since, in some instances, it overruled the advice of the occupational health physicians. This lead to internal communication problems, but also to dissatisfied heads of departments and individual HCWs, because the hospital board’s way of communication was too compelling. This barrier appeared to be the result of an attitude problem, with the board overruling the advice of other policy levels, without sufficiently trusting them to make the right decisions.

#### Barrier: communication (n = 4)

Communication as a barrier was experienced between different specialists working for the same hospital, which led to frustration and loss of time, as well as between the professionals and individual HCWs, where HCWs were less likely to agree with the new policy when the risks and necessity of vaccination were not clear.

### Barriers related to the guideline itself

#### Barrier: content of the guideline (n = 10)

Overall, there were seven specific elements of the measles guideline that raised questions or lead to discussion, a detailed description of which is presented in Table 2. These entail the following seven elements: justification of addressing measles; distinguishing immune from non-immune HCWs; the policy regarding visitors to high-risk departments; the definition of external personnel; risk categorization of various hospital of departments with respect to measles transmission; the definition of immunocompromised HCWs and, the advised level of hospital isolation of patients suspected with measles.

**Table 2:** Overview of the guideline-specific barriers obtained during the in-depth interviews

Element	Explanation
1. Justification of addressing measles	It was unclear why measles should be addressed in the hospital setting and why it poses a risk (n = 1)
2. Distinguishing immune from non-immune HCWs	It was not clear where to draw a line concerning immune or non-immune HCWs; the cut-off point at 1975 lead to discussion. Two participants mentioned that among the HCWs not protected according to the RIVM guideline, 65/72 HCWs who had their antibody levels tested actually were immune to measles (n = 4)
3. Visitors of high-risk departments	Four participants indicated to have had some discussion about how to deal with visitors of high-risk departments. The guideline does not discuss this aspect of infection prevention. (n = 4)
4. External personnel	The guideline was not clear on how to deal with external personnel, such as midwives. Since external personnel are not included in the hospital database in many cases, there is a risk of accidentally excluding them from the new policy (n = 2)
5. Risk estimation departments	The guideline should be more specific about making estimations of the risks different departments face, thereby enabling hospital professionals to better target their HCW vaccination policies (n = 2)
6. Immunocompromised HCW	In the guideline, immunocompromised HCWs are indicated to be at increased risk of severe course of disease after measles infection. However, it was unclear when one actually is immunocompromised (n = 2)
7. Isolation type	It was not clear why the RIVM guideline proposed strict isolation for measles cases as opposed to aerogenic, which is the standard form of isolation for measles. When it is not clear to the professionals, they indicate that they cannot convince their HCWs to follow protocol. Furthermore, since strict isolation is more expensive than aerogenic it was indicated that it should really be clarified (n = 4)

### Barriers related to contextual and social factors

Barriers related to contextual and social factors include external barriers related to finances and work pressure (n = 6), physical infrastructure (n = 6), national views on vaccination policies (n = 5), and timing of the MG (n = 7).

#### Barrier: finances and work pressure (n = 6)

Finances played a significant role in decision-making processes. Cost analyses determined which HCWs to target for vaccination, as well as how to implement the vaccination policy. One hospital decided not to check for immunity but vaccinated at once, rather than carry out laboratory testing because the latter was thought to be too expensive. Buying and administering the vaccine was done on cost of the hospital in all cases. Four hospitals organised the vaccination themselves, whereas others sent their HCWs to Public Health Services (PHS’s) to save time and money. Furthermore, one participant indicated that his/her workload increased because there was no money to hire staff working in occupational health services. One participant indicated that obtaining funds for prevention is difficult:

“That is always the case in our profession, […], you spend a lot of money to make sure that something doesn’t happen of which you cannot prove it ever would have happened.” [participant 5]

Handling the epidemic was characterised by the input of a large amount of man-hours, adding to the workload of six participants. One hospital was forced to hire extra staff to handle the workload.

#### Barrier: physical infrastructure (n = 6)

The physical infrastructure of the hospitals hampered the implementation of the guideline in several ways. First, the availability of digital databases to register immune status of HCWs was a significant barrier in three hospitals. There, databases were not in place at all, or are only able to check the immune status per HCW. It was not possible to obtain an overview of the (non-) immune HCWs. In other hospitals where these databases were in place, they were often not used to register immunity to measles because the system had “no space for this” [participant 8]. Secondly, following the guideline was hampered by the limited availability of isolation facilities in four hospitals. Participants believe the guideline should provide guidance on what to do in the face of insufficient capacity.

#### Barrier: national views on vaccination policies (n = 5)

Five participants considered national views on vaccination policies a barrier. Participants addressed wanting clear advice on a national level on what is allowed, both legally and ethically, when it comes to the vaccination of HCWs.

#### Overview of barriers encountered by ≤4 participants

For the implementation of the new policy, the specialists are dependent on the cooperation of the HCWs themselves, something that cannot be controlled (lack of outcome expectancy, n = 2). Inertia of previous practice was addressed by one participant who indicated that the routines of the infection preventionists sometimes clash with newly introduced policy changes, while three respondents mentioned unclear division of labour (n = 3) to be a barrier for the implementation, when teamwork was compromised. Two participants were afraid that HCWs would become tired of all the new disease policies and that if something serious would happen, they would not be followed as seriously anymore (n = 2). Three participants indicated that there were feelings of fear about how big the epidemic would be (n = 3). Two participants indicated that the measles epidemic was addressed on a small scale in their hospital (n = 2) and that their work therefore felt like “pioneering” [p. 4].

Participants considered the phrasing of the guideline to include too much jargon and in some instances it was written with insufficient urgency (n = 3). Jargon could be specific for the medical specialty, as well as for the infection prevention specialty; in some instances the guideline was written with insufficient urgency allowing for variation in the interpretation of the need for vaccination.

Hospital culture (n = 2) was mentioned as a contextual factor that influences uptake of vaccination policies. Is not easy for HCWs to expose their immune status in religious communities to which fellow colleagues belong if religion does not allow vaccination (n = 2). Furthermore, the need for vaccination only became clear to some HCWs once actual measles cases presented or when news about measles in the health care setting appeared (n = 2), as described by participant 4:

“People are emotional creatures and when it comes to it… [they will want to vaccinate]. In my experience, this goes for nurses even more than for doctors.” [participant 4]

The presence of multiple guidelines posed a barrier (n = 3), since the WIP and RIVM guideline differed initially with respect to urgency as well as isolation protocols in their respective guidelines. Physical infrastructure posed a barrier since two participants indicated that some aspects of the guideline could not be followed when the hospital lacks a clear distinction between high- and low risk departments. Timing as a barrier was addressed several times. First of all, timeliness of the guideline was a barrier encountered by two participants because it arrived after their policy had already been formulated and communicated. However, for most hospitals the guideline arrived on time. Secondly, one hospital indicated that the measles epidemic occurred simultaneously with cases of Methicillin-Resistant Staphylococcus Aureus (MRSA), forcing professionals to divide their time and attention between the diseases. Thirdly, participants were involved in bringing together different specialists in a task force or outbreak management team. These groups included: microbiologists, occupational health physicians, communication experts, neonatologists and paediatricians. Bringing together these work groups was negatively influenced when specialists were not physically present in the hospital (due to vacation), which forced the participants to communicate by telephone or e-mail (n = 2). This made communication less efficient due to significant delays in response time and lack of face-to-face contact. One hospital does not have an infection prevention committee during the holiday period, while another participant indicated to have been solely responsible for handling the epidemic because his/her colleagues were away.

## Discussion

The objective of this research was to identify barriers that Dutch hospital professionals encountered during the implementation of the 2013 policy guideline on measles. In general, this study showed that the measles guideline was considered practical and provided guidance during outbreak situations. However, 17 different barriers were found to hamper its implementation. Several new barriers were identified in addition to those that had been hypothesised to be of importance based on literature.

Of the 7 barriers from the Cabana framework [18] the majority hampered implementation of the measles guideline, with the exception of “lack of awareness”, “lack of familiarity” and “lack of self-efficacy”. An unexpected finding was that the lack of agreement relied on motivation issues and led to manifest resistance to vaccination. While exploring reasons for resistance to HCW immunization, a variety of social factors were mentioned (including religious beliefs, ethical questions and fear of side effects). Thus, vaccine availability, disseminating the guideline and removal of the organizational barriers alone may not suffice to improve policy implementation. Increasing motivation, another important factor, will require interventions tailored to every single factor, within a multi-faceted approach. Preferably this should take place before the next outbreak as measles is a vaccine preventable disease and it is not likely that the attitude towards vaccinations will change significantly, given the strong beliefs and personal opinions exhibited by certain categories of HCW.

All barriers related to resources for implementation and cultural aspects of the organisation identified by Cahill et al. [19] were found to be of importance to participants of this research. These barriers include leadership style, communication, unclear division of labour, finances and work pressure, physical infrastructure of the organisation and hospital culture. Concerning hospital culture, this research showed in particular that certain religious beliefs, as part of culture in general, can specifically affect guideline implementation. Finally, two out of four barriers identified by Timen et al. hampered implementation of the measles guideline: “Control measures are worded with insufficient urgency or definition” and “Crucial instructions within control measures (concerning isolation, diagnostics, and treatment) are not clear or easily identifiable for each profession” [20].

Barriers that emerged from this research, but not from literature specifically, can stem from guideline content itself, (evidence-based) decision making, can also be the result of insufficient human, financial, physical and/or technical resources, poor timing of guidance and implementation, lack of information, communication or clarity on all levels (national to individual). Moreover, although culture of the organisation was mentioned by Cahill et al. [19], this research showed that certain religious beliefs, an aspect of culture in general, can affect guideline implementation.

The conceptual model used in this research was found to adequately represent the different barriers encountered by hospital professionals during the implementation of the RIVM guideline. Much can be learned from the wide range of qualitative information gained from this research. These insights can be used to improve the implementation of similar guidelines in the future.

### Barriers related to knowledge and attitude

With regard to barriers related to knowledge and attitude, several changes can be made in future guidelines. First of all, it is important to be explicit about the risks associated with measles in the hospital setting, as well as the risks of not being vaccinated and the potential risks associated with vaccination. A clear guideline enhances understanding of issue(s) and promotes agreement on both the level of the individual HCW carrying out the policy and the professional handing down the directive, improving implementation. In order to increase measles vaccination uptake, it is important to incorporate advice on how to deal with HCWs who don’t comply with vaccination requests or who are reluctant to cooperate based on religious reasons. Second, in order to prevent professionals from feeling that their efforts are not worth the risks, it is important to provide as much detailed information as possible about the actual cases that occur during an outbreak. This evidence base must be specific for different regions or hospitals to enable hospital professionals to make informed decisions about whether to take (additional) measures during outbreak situations. Thirdly, since communication and teamwork were found to be important determinants of successful guideline implementation, the guideline could provide information on how best to determine where the responsibilities of different specialists lie with regard to the development and implementation of HCW vaccination policies. This will enable hospital professionals to work together efficiently without losing time deciding where these responsibilities lie. In addition, this could reduce friction between different departments that results from inertia of previous practice. Finally, since leadership is an important determinant of successful guideline implementation it is important to advise hospitals to involve the hospital board early on during outbreak situations. Having committed officials or leaders is a key condition for successful (top-down) policies [27]; therefore, early involvement of the hospital board could increase the uptake of the policy on both the level of individual HCWs and the department heads. The latter will reduce the chance they will oppose the policy for fear of increased work pressure or loss of autonomy and staff.

### Barriers related to characteristics of the guideline

With regard to barriers related to characteristics of the guideline, several changes can be made in future guidelines. First of all, all seven aspects of the guideline content that were not clear should be addressed in similar guidelines in the future. This implies that specific recommendations should be made on dealing with visitors to high-risk departments, external personnel and immunocompromised HCWs. Furthermore, the guideline should discuss self-reported immunity in more detail and better explain the rationale behind strict isolation and how to make risk assessments of hospital departments. The latter is particularly relevant for small hospitals, which do not always divide their hospital departments into high- and low risk departments. There, patients are distributed to a limited number of departments, thereby “mixing” high- and low risk patient categories. Secondly, it is important to phrase the guideline carefully, with sufficient urgency, while avoiding language that is too technical.

### Barriers related to contextual and social factors

The guideline should provide recommendations about what to do when confronted with insufficient hospital capacity, and when distinctions between high-risk and low-risk departments are unclear. Secondly, one of the crucial elements of the RIVM guideline recommends a good database to register individual and aggregate HCW immune status information. This study showed, however, that these databases are present in only a minority of hospitals and that current databases are not able to provide complete clarity of immune status to the hospital professionals. Therefore, it is pivotal that hospitals upgrade their databases to meet the challenges they face during outbreak situations, while guaranteeing HCW privacy. The need for digital human resource databases is underscored by research done by Bertin et al. [28]. All HCWs from the Cleveland Clinic, Ohio (United States), both with and without direct patient contact, were required to document their immune status through Intranet. The HCWs could indicate their vaccination status with regard to the annual influenza vaccination. The Intranet site was linked to a digital database, available on both the individual and department level. Data were kept confidential by making use of unique identification numbers. HCWs declining vaccination received an automatic message with information about the vaccination. After implementation of this new policy, the vaccination rate increased significantly (from 38 to 55 %) [28]. This digital method of obtaining HCW immune status, linked to digital databases, could not only increase policy uptake in hospitals by addressing individual HCWs, but could also enable heads of departments to take control of their department, thereby counteracting potential feelings of loss of autonomy. Thirdly, it is important that ethical and legal guidance is provided on a national level to decide which measures hospitals can take without compromising the rights of HCWs. This includes whether it is justified to make vaccination against measles obligatory upon employment and to register immune status of new HCWs. Since seven out of ten hospitals indicated they are looking into the possibilities for these measures, it is clear that a change in culture is visible. The importance of mandating vaccination becomes clear from a study conducted by Nowalk et al. [29], who assessed American hospitals with mandatory annual HCW influenza vaccination by comparing the effects of ‘having’ with ‘not having’ consequences attached to non-compliance with this vaccination. The researchers found that hospitals with consequences have vaccination rates of 86 %, whereas hospitals without consequences have rates of 67 %. Furthermore, having state laws regulate HCW vaccination for influenza was associated with nearly a threefold increase in rates for mandates with consequences compared to mandates without consequences [29]. The pros and cons of mandatory vaccination of HCWs should be taken into account in the future, in the light of the worldwide efforts to eradicate vaccine-preventable diseases.

### Strengths and limitations

This study has several strengths and has provided insights into vaccination policies in Dutch hospitals and the extent to which the MG has contributed to these policies. Participants belonged to different professions within infection prevention, thereby broadening the scope of this research through a wide variety of perspectives. Secondly, these professionals represented both academic and non-academic hospitals, located in the three different regions of the Netherlands, thereby increasing the external validity of this research. Thirdly, the fact that this research was conducted within a relative short period of time after the epidemic enhances the reliability of the respondents’ recall of participants’ experiences. However, this study has several limitations. First of all, it is unclear to what extent the Dutch setting can be generalised to other countries both in and outside of Europe. Second, thirteen hospitals were approached for participation, while only ten are included. The missing three hospitals that did not respond might differ in their positive and negative performances with regard to MG implementation. From unpublished research it can be concluded, however, that the included ten hospitals provide a good reflection of the Dutch hospitals. The current qualitative study was conducted simultaneously with a quantitative study that aimed to assess the uptake and adaptation of the RIVM guideline. The quantitative study provided a cross-sectional picture of guideline uptake, rather than explore in depth its performance. As part of this quantitative study a questionnaire with 70 questions was sent to infection prevention specialists of Dutch hospitals. It revealed that 68 participants (95.8 %) believed the guideline was written clearly or very clearly; 56 participants (81.2 %) indicated that the guideline fits with their situation; 51 participants (71.8 %) felt that the guideline can contribute to a reduced number of measles cases in the hospital and 64 participants (90,1 %) believed the guideline takes into account the wishes and preferences of HCWs either sufficiently or more than sufficiently. Furthermore, 27 (38.0 %) participants encountered some resistance, while 5 (7.0 %) met with a lot of resistance [unpublished observations, L. Fievez MD]. Since these findings show trends that correspond with the findings of this research, the sample of 12 participants can be considered a good reflection of the general population of professionals working in infection prevention. Thirdly, although recommendations for improvement are made, the authors acknowledge that several aspects of guideline implementation cannot be influenced. These include non-complying HCWs (e.g. due to personal beliefs or religion) and internal issues with implementing the policy due to for example vacation of infection prevention specialists or individual HCWs.

## Conclusions

The implementation of the MG in the healthcare setting was hampered by a total of 17 different barriers. The most important barriers related to knowledge and attitude reflect lack of agreement, issues related to evidence-based decision making and leadership. Barriers related to characteristics of the guideline are mostly due to unclear or missing content of the MG. Seven elements of the guideline were reason for discussion: the justification of addressing measles in the hospital setting; determining immune status based on self-report; dealing with visitors to high-risk departments; dealing with external personnel; making risk assessments for infection at different hospital departments; determining when an individual is immunocompromised and the rationale for following strict isolation protocol. For barriers related to contextual and social factors, the most prominent barriers relate to finances and work pressure, physical infrastructure and national views on vaccination policies.

The authors believe this study has provided valuable insights into the barriers infection prevention specialists encounter during the implementation of new policies concerning the vaccination of HCWs. Moreover, this study exposed the complexity and numerousness of barriers that are of importance when implementing vaccination policies in the hospital setting. In order to further increase knowledge in this area, further research is needed into the reasons underlying non-compliance of HCWs in hospitals and into the self-reporting of immune status.

## Recommendations

The authors make eight recommendations for future guidelines in the field of public health.

1. The guideline should be explicit about the risks associated with introduction and transmission of vaccine-preventable diseases in the hospital setting.
2. The guideline should incorporate advice on how to deal with HCWs who do not cooperate with vaccination policies (either due to non-response or resistance).
3. The guideline should provide advice on how to distinguish between the roles of different professionals during outbreak situations; i.e. which specialty is responsible for which part of the outbreak containment strategy. This includes the decision-making, the implementation and evaluation of the employed strategy.
4. A strategy should be designed on how to involve the hospital board early during outbreak situations.
5. The guideline should be phrased carefully, while paying attention to avoiding language that includes too much jargon or wording the recommendations with insufficient urgency.
6. The guideline should provide recommendations on what to do in the face of insufficient capacity and in the face of lacking clear distinctions between high-risk and low-risk departments (specifically in small hospitals).
7. The guideline should clearly advise professionals to use digital databases to register HCW immune status.
8. Region specific information on the number of disease cases should be provided during outbreak situations, to allow hospital professionals to make informed decisions about the course of their policies.

## Abbreviations

COREQ: Consolidated criteria for reporting qualitative research
FFP: filtering face piece
HCW: healthcare worker
HI: health inspectorate
MG: measles guideline
MMR: measles mumps rubella
PHS: Public Health Service
RIVM: National Institute for Public Health and the Environment
WIP: Working Party on Infection Prevention
WMO: Wet Medisch-wetenschappelijk Onderzoek met mensen (Medical Research Involving Human Subjects Act)
